# Mortality of Dairy Sheep during the Peri-Parturient Period: Results of a Field Investigation in Greece

**DOI:** 10.3390/ani11082172

**Published:** 2021-07-22

**Authors:** Antonis P. Politis, Natalia G. C. Vasileiou, Peter J. Cripps, Dimitra V. Liagka, Petros T. Boufis, Irene Valasi, Vasia S. Mavrogianni, George C. Fthenakis

**Affiliations:** 1 Veterinary Faculty, University of Thessaly, 43100 Karditsa, Greece; apolitis.vet@gmail.com (A.P.P.); peterjohncripps@gmail.com (P.J.C.); dliagka@vet.uth.gr (D.V.L.); pboufisvet@gmail.com (P.T.B.); evalasi@vet.uth.gr (I.V.); gcf@vet.uth.gr (G.C.F.); 2 Faculty of Animal Science, University of Thessaly, 41110 Larissa, Greece; vasileiounat@gmail.com

**Keywords:** death, dystocia, lambing, mastitis, metritis, post-partum, pregnancy toxaemia, respiratory infection, ruminal acidosis, sheep

## Abstract

**Simple Summary:**

The paper presents a field investigation into the death of ewes around the peri-parturient (lambing) period. The problem is financially important because, in such cases, the capital (i.e., the ewes) is lost along with the increased expenses incurred by farmers during gestation. The problem was found to occur sporadically. Most cases of death occurred before lambing. Pregnancy toxaemia, an important metabolic disease, was the clinical problem most often associated with peri-parturient death. Factors found to be significantly associated with occurrence of the problem included the average lambing rate, the season of the year during which the lambing period took place, and the number of animals in the flock.

**Abstract:**

Objectives of the study were (a) to investigate the incidence risk of peri-parturient mortality in dairy flocks in Greece, (b) to study when such mortality occurs in relation to lambing, (c) to identify clinical problems associated with the deaths, and (d) to evaluate potentially associated factors. The deaths of ewes during the peri-parturient period (10 days before to 7 days after lambing) were monitored in 60 flocks over two years. In the ewes that died, detailed post-mortems were performed. The incidence risk of peri-parturient deaths was 0.7%. The daily incidence rate of deaths before, at, and after lambing was 0.05, 0.04, and 0.03%, respectively. Clinical problems most frequently associated with peri-parturient deaths were pregnancy toxaemia, ruminal acidosis and post-partum genital problems. For the occurrence of peri-parturient deaths in flocks, the average lambing rate in the flocks was the only significant factor. Regarding deaths before or after lambing, the season during which lambing took place and the number of animals in the flock, respectively, were found to be significant. Most ewes (54.5%) that died at lambing (dystocia) were primigravidae.

## 1. Introduction

The peri-parturient period has been recognised as a particularly dangerous period in the life of animals. In cows, there is with an increased risk of death and culling due to serious disease [1]. Similar findings have been reported for sows, animals in which peri-parturient diseases (lactational failure, genital tract disorders) were found to account for the loss of approximately 25% of adult pigs [2], as well as for mares, animals in which peri-parturient haemorrhaging is a significant cause of illness and death [3]. In women, incidence of peri-parturient deaths can be as high as 0.45% in some countries [4], with haemorrhage, hypertensive disorders, abortion and sepsis considered to be the main clinical conditions associated with death [5].

Despite the above, there are very little data regarding the occurrence of deaths of ewes during the peri-parturient period, a problem initially described by Hindson and Winter [6]. More recently, Mavrogianni and Brozos [7] classified these deaths according to the time in relation to lambing (before, at, or after) and provided a detailed guide for the diagnostic approach that needs to be followed in such circumstances. Nevertheless, no detailed data are available internationally regarding the incidence, the time of death in relation to lambing and the factors potentially associated with the occurrence of death. Such data would have allowed a better appraisal of the problem and would have been useful for improving health management of sheep flocks. Relevant data for ewes have focused on the mortality rate of specific disorders that occur during that period; for example, Sobiraj [8] reported a 10% mortality among ewes with dystocia.

The objectives of the present study were (a) to investigate the incidence risk of peri-parturient mortality in a field investigation in flocks in Greece, (b) to study the time that such mortality occurs in relation to lambing, (c) to identify the clinical problems more frequently associated with the deaths and (d) to evaluate factors potentially associated with the peri-parturient deaths.

## 2. Materials and Methods

### 2.1. Flocks and Investigation Procedures

Sixty (60) dairy sheep flocks were included in the study, which was performed during two consecutive years, from August 2017 to July 2018 and from August 2018 to July 2019. In total, there were 13,418 and 13,599 ewes at the start of each of the two study years, respectively, in the 60 flocks. In accordance with the national procedures for animal identification, all ewes bore plastic ear-tags in both ears with a unique national registry number. All the flocks were located in the administrative region of Western Greece and maintained for dairy production; in most flocks, lambs were weaned at 45 to 60 days (although occasionally early weaning occurred at 20–25 days) and then ewes were milked for a period of 6 to 8 months. Flocks in the study came from small-sized family farms to large commercial farms, with 50 to 812 ewes therein. Data regarding management of the flocks, available to the attending veterinarian as part of the standard monitoring and veterinary care of the flocks were considered (Appendix A and Appendix B).

During the study period, all deaths in each of the flocks were recorded. All flocks were under the veterinary care of the main author (APP), who visited them regularly (every 3 to 4 days during the lambing period and once fortnightly otherwise), as well as outside that schedule in cases of emergency. The protocols of the study were approved by the academic board of the Veterinary Faculty of the University of Thessaly, meeting 37/02.11.16.

At the individual animal level, the peri-parturient period was defined from 10 days before to 7 days after lambing (i.e., a total of 18 days). For all the ewes that died during that period, as reported by the respective farmers, a detailed post-mortem examination was performed by the main author (APP), who was also responsible for the veterinary care of the flocks. The time of death in relation to lambing was established. For this, the farmer provided the initial relevant information, which was subsequently confirmed by a post-mortem examination of the genital tract [6]. If the cervix was closed and the uterus was gravid, then the ewe was considered to have died before lambing. If the cervix was open with presence of a dead embryo or fetal remains in the uterus or the genital tract, then death was considered to have occurred at the time of lambing. In the absence of embryo(s) within the uterus, the ewe was considered to have recently lambed or aborted; in that case, the cervix could be found open or, after the third to fourth day after lambing, closed, although material indicative of a recent gestation would have been evident in the uterus [9].

During the post-mortem examination, the following were evaluated, as recommended by Mavrogianni and Brozos [7].

- If the ewe died before lambing: body condition, liver, perirenal and peritoneal fat, urine (sampling for measurement of ketone bodies), aqueous humour (sampling for measurement of calcium (only within 24 h of death)), placenta and condition of the foetus(es), and other organs;
- If the ewe died at lambing: opening of the cervix, foetus(es), placenta, genital tract, other organs, and aqueous humour (sampling for measurement of calcium (only within 24 h of death));
- If the ewe died after lambing: genital tract, subcutaneous tissues, aqueous humour (sampling for measurement of calcium (only within 24 h of death)), mammary glands, and other organs.

Following the post-mortem examination and based on their results, relevant and appropriate examinations were also performed in the other animals of the flock and appropriate samples were collected, in order to establish a diagnosis of the prevailing problem at flock level. Finally, a diagnosis was made of the clinical problem in the ewe.

### 2.2. Data Management and Statistical Analysis

Data were entered into Microsoft Excel and analyzed using SPSS v. 21 (IBM Analytics, Armonk, NY, USA). A basic descriptive analysis was performed and exact binomial confidence intervals (CIs) were obtained.

In relation to the time of occurrence within each year of the study, deaths were classified as occurring before, during or after the peri-parturient period. Deaths within the period were classified as occurred before, at or after lambing by applying the above criteria.

For estimating the incidence rates and risks, the exact number of ewes that entered each study year, and the exact number of deaths were known. The number of animals that left the flock (i.e., culled or sold) was believed to be very low (<1%), but it was not recorded or included in the calculations. Incidence rates and risks for any time period were calculated by subtracting the cumulative sum of ewes that had died before that time period from the number that had started the year. The annual incidence risk of deaths or of peri-parturient deaths for each year of the study was defined as the proportion of ewes that died among those at risk. Then, an average incidence risk was calculated as the mean of the two annual incidence risks obtained. The incidence rates of peri-parturient deaths in each of the three components of the period (before, at, after lambing) were compared after taking into account the different lengths of each of these three components (i.e., 10, 1, 7 days, respectively, for a total of 18 days) by using a Poisson regression for comparison of events per sheep-day at risk.

Fifteen (15) variables were evaluated for potential association with peri-parturient deaths (Appendix A). For each of these variables, categories were created according to the farmers’ answers. The outcomes of “death before/at/after lambing” were considered. Exact binomial CI were obtained. Initially, the importance of predictors was assessed by using a cross-tabulation with Pearson’s chi-square test and with simple logistic regression without random effects. Subsequently, multivariable models were created using mixed-effects logistic regression with flocks as the random effect, and initially offering to the model all variables, which achieved a significance of *p* < 0.2 in the univariable analysis (Table S1). Variables were removed from the initial model by backwards elimination. The *p* value of removal of a variable was assessed by the likelihood ratio test, and for those with a *p* value of >0.2 the variable with the largest probability was removed. This process was repeated until no variable could be removed with a *p* value of >0.2. The variables required for the final multivariable model in each occasion are shown in Table S1. After performing the analyses as detailed above with the results of both years of the study considered together, the analyses were repeated with the results of each year of the study considered separately.

Finally, the outcomes “occurrence of peri-parturient deaths in at least one year of the study” and “occurrence of peri-parturient deaths in both years of the study” (i.e., independently of the time of death in relation to lambing) were considered. The above methodology was also employed for these analyses. The variables required for the final multivariate model for each occasion are shown in Table S1.

In all analyses, statistical significance was defined at *p* < 0.05.

## 3. Results

### 3.1. Incidence Risk of Peri-Parturient Deaths in the Sheep Flocks

A total of 345 deaths of adult ewes were recorded during the two years of the study. Specifically, 160 deaths occurred in the first and 185 deaths in the second, and the respective annual incidence risks were 1.2% (95% CI: 1.0–1.4%) and 1.4% (95% CI: 1.2–1.6%) (*p* = 0.22 between the two years); hence, the average incidence risk during the two years was 1.3%. There were 147 deaths outside the peri-parturient period: 79 before and 68 after it (*p* > 0.30 for all comparisons between deaths before or after the period and between years of the study).

A total of 198 deaths (57.4% of all deaths; 95% CI: 52.1–62.5%) occurred during the period. Specifically, 89 deaths occurred in the first and 109 deaths in the second year of the study and the respective annual incidence risks were 0.7% (95% CI: 0.6–0.8%) and 0.8% (95% CI: 0.7–1.0%) (*p* = 0.18 between the two years); hence, the average incidence risk during the two years was 0.7% (*p* < 0.0001 compared to deaths that occurred outside the peri-parturient period) (Table 1).

Among the flocks, the average incidence risk of peri-parturient deaths varied from 0.0 to 6.0% during the two years (*p* < 0.0001 between flocks) (Table S2). It varied from 0.0 to 8.8% in the first and 0.0 to 9.2% in the second year of the study. Peri-parturient deaths were noted in a total of 28 flocks (46.7, 95% CI: 34.6–59.1%). There was no significant difference in the proportion of flocks, in which peri-parturient deaths were noted in each year of the study: 25.0% (95% CI: 15.8–37.2%) and 38.3% (95% CI: 27.1–51.0%), respectively (*p* = 0.12), although in 10 flocks (16.7, 95% CI: 9.3–28.0%) deaths were noted during both years.

### 3.2. Time of Death in Relation to Lambing

**Figure 1:**
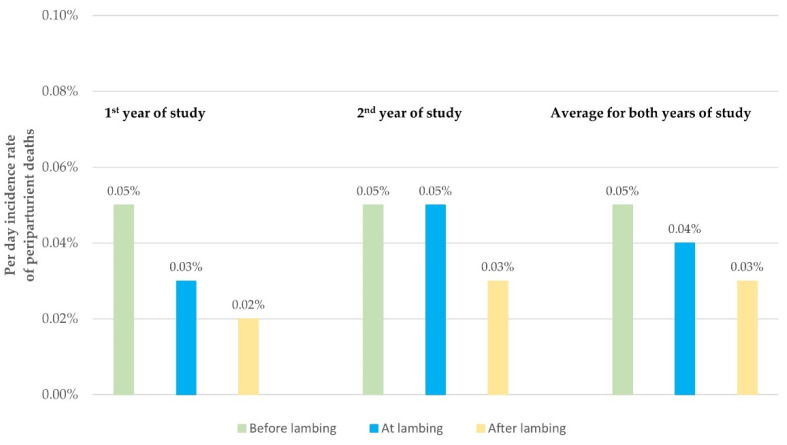
Daily incidence rate of peri-parturient deaths during a two-year study in 60 sheep flocks in Greece in relation to the time of lambing: green, before lambing (duration: 10 days); turquoise, at lambing (duration: 1 day), yellow, after lambing (duration: 7 days).

Of the 198 peri-parturient deaths, most (*n* = 135; 68.2%, 95% CI: 61.4–74.3%) occurred before lambing. Fewer deaths occurred at (*n* = 11; 5.6%, 95% CI: 3.1–9.7%) or after (*n* = 52; 26.3%, 95% CI: 20.6–32.8%) lambing. There was no significant difference in these proportions between the two years of the study (*p* > 0.10 for all comparisons) (Table S3). The per day incidence rate of deaths in each of the three components of the peri-parturient period (i.e., before, at, after lambing) was 0.05, 0.04 and 0.03%, respectively (*p* = 0.001 for the overall difference between the three components considered together, *p* < 0.001 for the difference in the incidence rate before and after lambing, *p* > 0.23 for the other comparisons) (Table 2, Figure 1).

Deaths before, at, and after lambing were noted in 24 (40.0%), 5 (8.3%) and 20 (33.3%) flocks, respectively (*p* = 0.0002). In 3 flocks (5.0%, 95% CI: 1.7–13.7%), deaths were noted during the same year in ewes before, at and after lambing.

### 3.3. Clinical Problems Associated with Peri-Parturient Deaths

**Figure 2:**
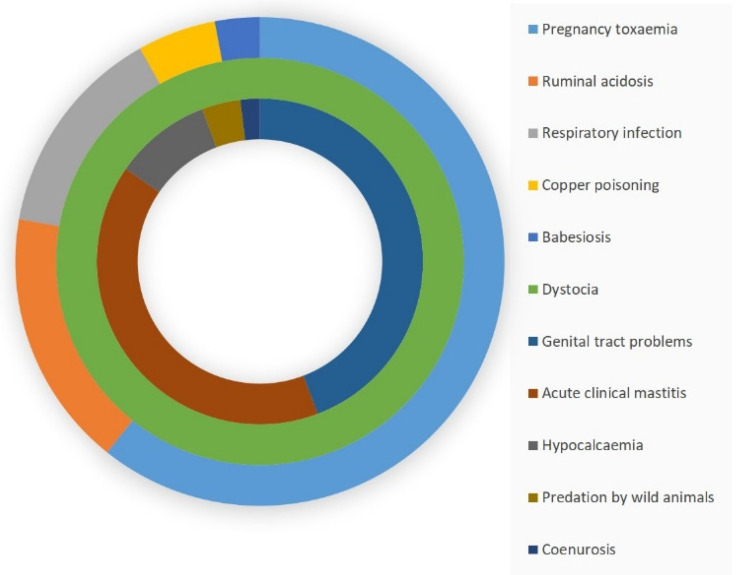
Ring-pie of the relative frequency of clinical problems associated with peri-parturient deaths in relation to the time of lambing: outer ring, associated with deaths before lambing (duration: 10 days); middle ring, associated with deaths at lambing (duration: 1 day); inner ring: associated with deaths after lambing (duration: 7 days).

Pregnancy toxaemia was the clinical problem most frequently associated with peri-parturient deaths (*n* = 82; 41.4% of all deaths, 60.7% of deaths before lambing). Other clinical problems were ruminal acidosis (*n* = 23; 11.6% of all deaths, 17.0% of deaths before lambing), genital tract problems (including genital injury, uterine prolapse, rectal prolapse, metritis) (*n* = 23; 11.6% of all deaths, 44.2% of deaths after lambing), acute clinical mastitis (*n* = 21; 10.6% of all deaths, 40.4% of deaths after lambing), respiratory infection (*n* = 19; 9.6% of all deaths, 14.1% of deaths before lambing) and dystocia (*n* = 11; 5.6% of all deaths, 100.0% of deaths at lambing) (Figure 2, Table S4). There was no significant difference between the two years of the study in the proportion of the various clinical problems associated with such deaths (*p* = 0.27).

Table 3 presents the numbers of flocks with various clinical problems associated with peri-parturient deaths. Again, pregnancy toxaemia occurred more often than any other problem. Related deaths were found in 19 flocks, and in 15 (78.9%) of these peri-parturient deaths associated with ruminal acidosis, dystocia, genital tract problems or acute clinical mastitis. Furthermore, in 6 of the 7 flocks (85.7%) in which ruminal acidosis was found, cases of pregnancy toxaemia were also recorded. Moreover, they were recorded in 7 of 11 flocks (63.6%) that had genital tract problems, in 7 of 11 flocks (63.6%) that had mastitis, and in 3 of 5 flocks (60.0%) that had dystocia (*p* = 0.72).

### 3.4. Age of Ewes That Died

**Figure 3:**
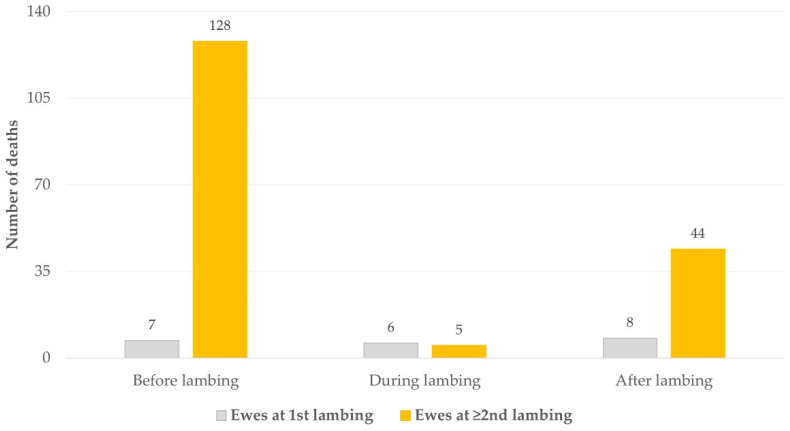
Frequency of peri-parturient deaths in relation to the time of lambing and the age of the ewes that died, during a two-year study in 60 sheep flocks in Greece (gray: ewes at 1st lambing; orange: ewes at ≥ 2nd lambing). The duration of the period before lambing was 10 days; at lambing, 1 day; after lambing, 7 days.

Of the 198 ewes that died during the peri-parturient period, 13 were primigravidae (7 died before and 6 at lambing) and 8 were primiparous; that is, 21 ewes (10.6% of all that died) had not lambed before (Figure 3). Of the 11 ewes that died in association with dystocia, 6 (54.5%) were primigravidae. The proportion of primigravidae ewes that died at lambing (54.5% of those that died at that stage) was significantly higher than of similar-aged ewes that died before or after lambing (8.0%) (*p* < 0.0001).

### 3.5. Factors Associated with Occurrence of Peri-parturient Deaths

With regard to the occurrence of peri-parturient deaths, a significant association was evident with the following two variables: the season when the lambing period took place (*p* = 0.008) and the average lambing rate at the flock level (*p* = 0.017). Among the variables included in the multivariate analysis, the average lambing rate at flock level turned out to be significant (*p* = 0.035), while a tendency was noted for the season during which the lambing period took place (*p* = 0.07) (Table 4).

With regard to deaths before lambing, a significant association was evident with 5 variables during the univariate analysis (Table S5). Among the variables included in the multivariate analysis, only the lambing season emerged to be a significant factor (*p* = 0.002).

With regard to deaths at lambing, a significant association was evident with 1 variable during the univariate analysis (Table S6). Among the variables included in the multivariate analysis, none was significant although a tendency for significance was seen for the lack of working staff in the flock (*p* = 0.059).

With regard to deaths after lambing, a significant association was evident with 3 variables during the univariate analysis (Table S7). Among the variables included in the multivariable analysis, only the number of animals in the flock was a significant factor (*p* = 0.024).

Details of the results of the multivariate analyses are in Table 5. When the analyses were repeated with the results of each year of the study considered separately, the findings were generally similar (Table S8).

## 4. Discussion

This study explored in detail, for the first time internationally, the death of ewes during the peri-parturient period. Hindson and Winter [6] and Mavrogianni and Brozos [7] were the first to describe the problem; however, those reports were based on the clinical experience of the authors rather than on data from organised field work. In contrast, the present paper presents an extensive study performed in 60 flocks of dairy sheep over two successive years. Peri-parturient mortality has a low incidence and performance of the study over two years provided helpful information on the sporadic occurrence of the problem from year to year, as well as allowing a more thorough exploration of possible risk factors.

First, the findings confirmed that peri-parturient deaths accounted for most deaths of adult ewes: almost 60% of the deaths in the study. Further, the findings indicated that the problem had a sporadic occurrence. The incidence risk was <1.0% and the problem was recorded in only a few flocks during both years.

The low incidence of peri-parturient mortality should not undermine its importance for sheep flocks. Peri-parturient deaths have a significant impact on the flock. To reach the peri-parturient period, the ewes were subjected to an intensive preparation, while high input from farmers was required as well. This input included the use of infrastructure, feeding and intensive labour availability, as well as extended veterinary care for pregnancy diagnosis, and administration of vaccines and pharmaceuticals [10]; hence, if the ewe died there would be no return on investment. In such cases, the capital investment (i.e., ewes) perishes along with all the work in anticipation for the lambing; these expenses can often add up to the value of a ewe. The importance is similar in dairy- and meat-production systems. In a recent study of ewe wastage in meat production systems in New Zealand, a much higher removal rate from the flocks from mating to lambing were noted [11] compared to this study. This can be explained if one considers that in dairy systems, ewes would be milked until 30 to 45 days before the expected lambing ( i.e., farmers would provide care and infrequently cull animals until after the end of the lambing period).

In this study, no records were available regarding the ewes that had been culled. Inevitably, this possibly led to a slight over-estimate of the number of ewes and ewe-days at risk, so the estimated peri-parturient mortality rates are likely to be slightly below their true values. Because of the husbandry systems used in these dairy flocks, very few animals were culled. Our estimates were indeed very close to the true values because (a) the pregnancy rate is normally >98% due to the extended breeding season, (b) ewes were milked until 30–40 days before the expected start of the lambing period; and (c) the ewes were often kept until the end of the reproductive year when there is a market for their meat.

Among peri-parturient deaths, the highest incidence rate was noted before lambing and was aligned with the clinical conditions associated with the deaths. The lack of any statistical difference with the incidence rate at lambing was in partial accord with the previous suggestions of Hindson and Winter [6] and Mavrogianni and Brozos [7], who indicated that most cases of peri-parturient deaths occurred at lambing time.

Pregnancy toxaemia, the clinical condition most commonly associated with death, was the most important metabolic disease of sheep and occurred frequently in all flocks (up to 10% in undernourished flocks [12]). The disease can be fatal, especially if veterinary intervention is delayed. Another clinical condition often associated with the death of affected ewes was ruminal acidosis, which can be acute and result to death over the course of hours [13,14]. These two conditions, cumulatively, accounted for over 50% of all deaths during the peri-parturient period. The last stage of gestation is particularly demanding metabolically; hence, the energy requirements of pregnant ewes increases, as the end of pregnancy approaches. It is thus recommended that the nutritional regime of pregnant ewes be progressively modified, with energy provisions to accommodate the increased requirements of the ewes [10]. Farmers may not always perform the necessary changes in the feeding regime in a timely manner, which can lead to pregnancy toxaemia, depending on various factors [15]. As farmers see the problem developing, they would realise the necessity to increased energy and may attempt to rectify the problem by providing increased amounts of cereals, which can then lead to acidosis (in 6 of 7 flocks with deaths associated with ruminal acidosis, deaths associated with pregnancy toxaemia were also recorded). The acute nature of these two situations is shown by the high mean number of ewes that died in each flock.

In winter due to the low temperatures and increased precipitation, pregnant ewes have increased metabolic requirements [16]. These can further predispose them to pregnancy toxaemia. Indeed, in a recent report from Australia, increased ewe mortality was reported after a season of high precipitation [17]. Moreover, other situations occurring during the winter may also play a role; for example, adverse weather conditions may hinder veterinary access to farms. All these contributed to the emergence of the association of winter with a death before lambing.

Dystocia was the only clinical problem associated with death at the time of lambing. The lack of farm staff was found to contribute, to some extent, to this; this was reasonable, as many lambings might occur during the night, and personnel assistance is crucial for dealing with such cases. Furthermore, most such cases occurred in primigravidae ewes, in which foeto–maternal disproportion occurs more frequently [18]; in such cases, the genital tract may not be fully developed, especially if the ewe-lambs were mated too young [19].

Finally, post-partum genital disorders and clinical mastitis were the problems associated with death of ewes after lambing. Many of the genital disorders might have been the sequelae of dystocia that were dealt with inappropriately (e.g., uterine rupture [19]). Obstetrical manipulations without maintaining aseptic conditions can lead to metritis [20], and uterine and rectal prolapse are often the consequences of repeated straining by parturient ewes [21]. It is also noteworthy that pregnancy toxaemia can often lead to post-partum problems of the genital tract [9,22] and has been associated with the development of clinical mastitis during the first week post-partum [23]. Hence, one can postulate that, in some flocks, ewes with pregnancy toxaemia that had successful veterinary attendance, later developed problems post-partum, as described in previous studies [9,22]. It is also possible, given the immunocompromise present during that period, that the affected ewes were unable to counteract a mammary infection, and so suffered acute clinical mastitis and death [24,25]. Moreover, in animals with increased concentrations of *β*-hydroxybutyrate (which occurs in pregnancy toxaemia), Galvao et al. [26] found reduced blood neutrophil numbers, an issue that can clearly predispose a ewe to mastitis, given the importance of neutrophils in the defence mechanisms of the mammary gland [27].

It was interesting to note that while differing predictors were identified for deaths before or after lambing, another one, the average lambing rate at flock level, was found to be significant for the occurrence of deaths independent of the stage of lambing. Higher lambing rates at flock level (over 1.5) were found to be associated with the increased risk of peri-parturient deaths. It is possible that the increased lambing rate could connect all the issues mentioned above. The large number of foetuses predisposes ewes to pregnancy toxaemia [12] and increases the risk of dystocia [19]; furthermore, an increased litter has been positively associated with the development of mastitis [28,29] and metritis [30,31].

## 5. Conclusions

Peri-parturient deaths were found to occur sporadically and most cases occurred 10 days prior to lambing; pregnancy toxaemia was the clinical problem most frequently associated with peri-parturient mortality. Factors found to have a significant association with occurrence of peri-parturient mortality included the average lambing rate at flock level (increased risk with average lambing rate >1.5), the season of the year during which the lambing period took place (highest risk during the winter), and the number of animals in the flock (highest risk in the flock with ≤200 ewes).

## Figures and Tables

**Table 1:** Frequency and incidence risk of deaths of adult ewes during a two-year study in 60 sheep flocks in Greece.

1st Year of the Study				2nd Year of the Study				Both Years	
Ewes	Deaths Before the Pp Period ^1^	Deaths During the Pp Period	Deaths After the Pp Period	Ewes	Deaths Before the Pp Period	Deaths During the Pp Period	Deaths After the Pp Period	All Deaths	Deaths During the Pp Period
*n*									
13,418	40	89	31	13,599	39	109	37	345	198
Incidence risks									
	0.3% ^a^	0.7% ^a,b^	0.2% ^b^		0.3% ^c^	0.8% ^c,d^	0.3% ^d^	1.3%	0.7%

^1^ Pp: peri-parturient period. ^a–d^ *p* < 0.001 for comparisons between incidence risks with similar superscripts.

**Table 2:** Incidence rate of deaths in the three components of the peri-parturient period ^1^ during a two-year study in 60 sheep flocks in Greece (Poisson regression).

Components of the Peri-Parturient Period	Incidence Rate ^2^	Incidence Rate Ratio ^3^ (95% Confidence Intervals)	*p*-Value
			0.001
Before lambing (duration: 10 days)	0.05%	1.807 (1.313–2.489)	<0.001
At lambing (duration: 1 day)	0.04%	1.480 (0.772–2.837)	0.237
After lambing (duration: 7 days)	0.03%	reference	

^1^ these deaths refer only to the 18-day long peri-parturient period; ^2^ incidence rate per sheep-day at risk; ^3^ incidence rate ratios calculated against the lowest incidence rate.

**Table 3:** Number of flocks in which the clinical problems associated with peri-parturient deaths ^1^, were recorded during a two-year study in 60 sheep flocks in Greece.

Clinical Problems	Number (Proportion) of Flocks	Mean Number of Ewes That Died Per Flock
Pregnancy toxaemia	19 (31.7%)	4.3
Acute clinical mastitis	11 (18.3%)	1.9
Genital tract problems	11 (18.3%)	2.1
Respiratory infection	7 (11.7%)	2.7
Ruminal acidosis	7 (11.7%)	3.3
Dystocia	5 (8.3%)	2.2
Hypocalcaemia	4 (6.7%)	1.3
Copper poisoning	2 (3.3%)	3.5
Predation by wild animals	2 (3.3%)	1.0
Babesiosis	1 (1.7%)	4.0
Coenurosis	1 (1.7%)	1.0

^1^ these deaths refer only to the 18-day long peri-parturient period.

**Table 4:** Results of multivariate analysis for associations with occurrence ^1^ of peri-parturient deaths in at least one year of a two-year study in 60 sheep flock in Greece.

Variable	Proportion	Odds Ratio ^1^ (95% Confidence Intervals)	*p*-Value
Average lambing rate at flock level			0.035
≤1.5 (*n* = 45)	37.8%	reference	
>1.5 (*n* = 15)	73.3%	4.529 (1.243–16.510)	0.022

^1^ these deaths refer only to the 18-day long peri-parturient period.

**Table 5:** Results of multivariable analysis for associations with peri-parturient deaths ^1^ during a two-year study in 60 sheep flock in Greece.

Variable	Incidence Risk	Odds Ratio ^2^ (95% Confidence Intervals)	*p*-Value
Occurrence of Deaths: Before Lambing			
Season of the year during which the lambing period took place			
Winter (*n* = 5)	1.3% (18/1389) ^3^	3.088 (1.560–6.111)	0.002
Spring (*n* = 0)	n/a	n/a	0.001
Summer (*n* = 19)	0.7% (33/4589)	2.359 (1.383–4.022)	n/a
Autumn (*n* = 36)	0.3% (23/7513)	reference	0.002
Occurrence of Deaths: After Lambing			
Number of animals in the flock			
≤200 ewes (*n* = 38)	0.4% (18/5092)	3.990 (1.174–13.555)	0.024
200–500 ewes (*n* = 17)	0.2% (8/4944)	1.829 (0.483–6.876)	0.027
>500 ewes (*n* = 5)	0.1% (3/3377)	reference	0.375

^1^ these deaths refer only to the 18-day long peri-parturient period; ^2^ odds ratios calculated against the lowest prevalence associations of the variables; ^3^ n/m: number of ewes that died the respective component of the peri-parturient period/number of ewes at risk at the start of the respective component of the peri-parturient period (numbers averaged per flock from results of the two years of the study).

## Data Availability

Most data presented in this study are in the Supplementary Materials. The remaining data are available on request from the corresponding author. The data are not publicly available as they form part of the PhD thesis of the first author, which has not yet been examined, approved and uploaded in the official depository of Ph.D. Thesis from Greek Universities.

## Supplementary Materials

The following are available online at https://www.mdpi.com/article/10.3390/ani11082172/s1, Table S1: Details of multivariate models employed for the evaluation of the occurrence of peri-parturient deaths in sheep flocks in Greece. Table S2: Incidence risk of peri-parturient deaths in each of 60 sheep flocks during a two-year study in Greece. Table S3: Frequency of peri-parturient deaths in relation to the time of lambing, during a two-year study in 60 sheep flocks in Greece. Table S4: Frequency of clinical problems associated with peri-parturient deaths, in relation to the time of parturition during a two-year study in 60 sheep flocks in Greece. Table S5: Incidence risk of deaths of ewes during the peri-parturient period (before lambing) during a two-year study of 60 sheep flocks in Greece, in accord with factors (*n* = 15) evaluated for potential association with these deaths. Table S6: Incidence risk of deaths of ewes during the peri-parturient period (at lambing) during a two-year study in 60 sheep farms in Greece, in accord with factors (*n* = 15) evaluated for potential association with these deaths. Table S7: Incidence risk of deaths of ewes during the peri-parturient period (after lambing) during a two-year study in 60 sheep farms in Greece, in accord with factors (*n* = 15) evaluated for potential association with these deaths. Table S8: Summary of results of the analysis for associations with peri-parturient deaths, with the results of each year of the study considered separately.

## Appendix A

**Table A1:** Variables (*n* = 15; flock-level factors) evaluated for potential association with peri-parturient deaths in 60 sheep flocks in Greece.

Variables (*n* = 15)
Management system applied in the flock (description according to EFSA classification ^1^)
Season of the year during which the lambing period took place (autumn/winter/spring/summer)
Number of animals in the flock (no.)
Breed of ewes (Greek pure-breed animals/imported pure-breed animals/crossbreds)
Average lambing rate at flock level (no.)
Control or reproduction (yes with intravaginal use of progestogen sponges/yes with administration of melatonin implants/no)
Feed origin (homemade/commercial)
Inclusion of roughage into the diet (yes/no)
Intramammary administration of antibiotics at the end of the previous lactation period (yes/no)
Nutritional modifications performed during the last stage of pregnancy (yes/no)
Administration of anthelmintics at the end of pregnancy (yes/no)
Administration of anti-clostridial vaccination at the end of pregnancy (yes/no)
Administration of anti-mastitis vaccination at the end of pregnancy (yes/no)
Experience of the farmer (years)
Presence of working staff in the farm (yes/no)

^1^ European Food Safety Authority. Scientific opinion on the welfare risks related to the farming of sheep for wool, meat and milk production. *EFSA J.* **2014**, *12*, 3933–4060.

## Appendix B

**Table A2:** Characteristics of the variables (*n* = 15; flock-level factors) evaluated for potential association with peri-parturient deaths in 60 sheep flocks in Greece.

Management system applied in the flock					
Intensive		Semi-intensive		Semi-extensive or extensive	
*n* = 13		*n* = 12		*n* = 35	
**Season of the year during which the lambing period took place**					
Winter	Spring		Summer		Autumn
*n* = 5	*n* = 0		*n* = 19		*n* = 36
**Number of animals in the flocks**					
≤ 200 ewes		200–500 ewes		> 500 ewes	
*n* = 38		*n* = 17		*n* = 5	
**Breed of ewes**					
Greek pure-breed animals		Imported pure-breed animals		Crossbreds	
*n* = 15		*n* = 18		*n* = 27	
**Average number of lambs born per ewe at flock level**					
≤ 1.5			> 1.5		
*n* = 45			*n* = 15		
**Control of reproduction**					
Intravaginal use of progestogen sponges		Administration of melatonin implants		No	
*n* = 13		*n* = 9		*n* = 38	
**Feed origin**					
Homemade			Commercial		
*n* = 26			*n* = 34		
**Inclusion of roughage into the diet**					
Yes			No		
*n* = 58			*n* = 2		
**Intramammary administration of antibiotics at the end of the previous lactation period**					
Yes			No		
*n* = 21			*n* = 39		
**Nutritional modifications performed during the last stage of pregnancy**					
Yes			No		
*n* = 28			*n* = 32		
**Administration of anthelmintics at the end of pregnancy**					
Yes			No		
*n* = 60			*n* = 0		
**Administration of anti-clostridial vaccination at the end of pregnancy**					
Yes			No		
*n* = 60			*n* = 0		
**Administration of anti-mastitis vaccination at the end of pregnancy**					
Yes			No		
*n* = 25			*n* = 35		
**Experience of the farmer**					
0–10 years		11–20 years		> 20 years	
*n* = 13		*n* = 30		*n* = 17	
**Presence of working staff in the farm**					
Yes			No		
*n* = 39			*n* = 21		
