# Supplementary material for: Mortality of Dairy Sheep during the Peri-Parturient Period: Results of a Field Investigation in Greece
Source: Animals (Basel). 2021 Jul 22;11(8):2172. doi: 10.3390/ani11082172 (PMC8388358; doi:10.3390/ani11082172)
Supplement: Supplementary file 1 [file animals-11-02172-s001.zip › animals-1295324-supplementary.pdf]

animals

MDPI

Supplementary Materials

Mortality of dairy sheep during the peri-parturient period: results of a field investigation in Greece

Antonis P. Politis [1], Natalia G.C. Vasileiou [2], Peter J. Cripps [1], Dimitra V. Liagka [1], Petros T. Boufis [1], Irene Valasi [1], Vasia S. Mavrogianni [1,*] and George C. Fthenakis [1]

[1] Veterinary Faculty, University of Thessaly, 43100 Karditsa, Greece; apolitis.vet@gmail.com (A.P.P.); peterjohncripps@gmail.com (P.J.C.); dliagka@vet.uth.gr (D.V.L.); pboufisvet@gmail.com (P.T.B.); evalasi@vet.uth.gr (I.V.); gcf@vet.uth.gr (G.C.F.)
[2] Faculty of Animal Science, University of Thessaly, 41110 Larissa, Greece; vasileiounat@gmail.com
* Correspondence: vmavrog@vet.uth.gr

Table S1. Details of multivariable models employed for the evaluation of the occurrence of peri-parturient deaths in sheep flocks in Greece.

Outcome	Variables offered to the multivariable models (n)	Variables required in the final models
Death before lambing	5	(a) season of the year during which the lambing period took place, (b) number of animals in the flock
Death at lambing	4	(a) breed of ewes, (b) presence of working staff in the farm
Death after lambing	6	(a) number of animals in the flock, (b) breed of ewes, (c) control or reproduction, (d) nutritional modifications performed during the last stage of pregnancy, (e) experience of the farmer
Occurrence of peri-parturient deaths		(a) season of the year during which the lambing period took place, (b) average lambing rate at flock level, (c) inclusion of roughage into the diet
Occurrence of peri-parturient deaths in both years of the study		(a) experience of the farmer, (b) presence of working staff in the farm

Table S2. Incidence risk of peri-parturient deaths in each of 60 sheep flocks during a two-year study in Greece.

Farm reference no.	1st year of the study	2nd year of the study	Both years
1	1.32%	0.00%	0.66%
2	2.00%	5.00%	3.50%
3	0.00%	0.00%	0.00%
4	0.00%	0.00%	0.00%
5	0.00%	0.00%	0.00%
6	0.00%	1.80%	0.90%
7	0.00%	0.75%	0.38%
8	3.35%	3.28%	3.32%
9	0.00%	0.00%	0.00%
10	0.00%	0.00%	0.00%
11	0.00%	0.00%	0.00%
12	0.00%	0.00%	0.00%
13	0.00%	1.56%	0.78%
14	0.00%	0.00%	0.00%
15	0.00%	0.00%	0.00%
16	0.00%	0.00%	0.00%
17	0.00%	0.00%	0.00%
18	0.00%	0.95%	0.47%
19	0.00%	0.00%	0.00%
20	0.00%	0.00%	0.00%
21	1.04%	1.02%	1.03%
22	0.00%	1.31%	0.65%
23	0.00%	2.63%	1.32%
24	0.00%	4.08%	2.04%
25	1.47%	0.47%	0.97%
26	1.33%	2.07%	1.70%
27	0.00%	0.00%	0.00%
28	3.00%	0.00%	1.50%
29	0.00%	0.00%	0.00%
30	0.00%	1.14%	0.57%
31	6.29%	0.00%	3.15%
32	0.00%	0.00%	0.00%
33	0.00%	0.00%	0.00%
34	0.00%	0.00%	0.00%
35	0.00%	0.00%	0.00%
36	7.22%	0.00%	3.61%
37	0.00%	0.00%	0.00%
38	0.00%	0.00%	0.00%
39	0.00%	0.00%	0.00%
40	0.00%	0.00%	0.00%
41	3.39%	3.13%	3.26%
42	0.00%	0.00%	0.00%
43	1.83%	2.28%	2.06%
44	0.00%	1.49%	0.74%
45	0.00%	0.00%	0.00%
46	0.00%	0.00%	0.00%
47	0.00%	0.00%	0.00%
48	0.00%	0.00%	0.00%
49	0.00%	4.46%	2.23%
50	0.00%	0.00%	0.00%
51	8.84%	3.19%	6.01%
52	6.47%	2.87%	4.67%
53	0.00%	3.90%	1.95%
54	0.00%	0.00%	0.00%
55	0.00%	1.31%	0.65%

56	2.44%	0.00%	1.22%
57	0.00%	9.17%	4.58%
58	1.23%	1.75%	1.49%
59	0.00%	0.00%	0.00%
60	0.00%	0.00%	0.00%

Table S3. Frequency [1] of peri-parturient deaths in relation to the time of lambing, during a two-year study in 60 sheep flocks in Greece.

1st year of the study			2nd year of the study			Both years		
Time of death in relation to lambing								
Before	At	After	Before	At	After	Before	At	After
n								
	Total = 89			Total = 109			Total = 198	
65	4	20	70	7	32	135	11	52
Proportion of peri-parturient deaths within the respective period								
73.0%	4.5%	22.5%	64.2%	6.4%	29.4%	68.2%	5.5%	26.3%

[1] The figures refer only to deaths that occurred within the 18-day long peri-parturient period.

Table S4. Frequency [1] of clinical problems associated with peri-parturient deaths, in relation to the time of parturition during a two-year study in 60 sheep flocks in Greece.

Clinical problems	Time of death in relation to lambing		
	**Before**	**At**	**After**
Pregnancy toxaemia	82		
Ruminal acidosis	23		
Genital tract problems (genital injury, uterine prolapse, rectal prolapse, metritis)			23
Acute clinical mastitis			21
Respiratory infections	19		
Dystocia		11	
Copper poisoning	7		
Hypocalcaemia			5
Babesiosis	4		
Predation by wild animals			2
Coenurosis			1
Total	135	11	52

[1] The figures refer only to deaths that occurred within the 18-day long peri-parturient period.

Table S5. Incidence risk of deaths of ewes during the 'before lambing' component of the peri-parturient period during a two-year study in 60 sheep farms in Greece, in accord with factors (*n* = 15) evaluated for potential association with these deaths.

**Management system applied in the flock**				
Intensive (*n* = 13)	Semi-intensive (*n* = 12)	Semi-extensive or extensive (*n* = 35)		*p*
0.9%	0.3%	0.5%		0.002
**Season of the year during which the lambing period took place**				
Winter (*n* = 5)	Spring (*n* = 0)	Summer (*n* = 19)	Autumn (*n* = 36)	*p*
1.3%	n/a	0.7%	0.3%	<0.0001
**Number of animals in the flock**				
≤200 ewes (*n* = 38)	200–500 ewes (*n* = 17)	>500 ewes (*n* = 5)		*p*
0.7%	0.5%	0.3%		0.034
**Breed of ewes**				
Greek pure-breed animals (*n* = 15)	Imported pure-breed animals (*n* = 18)	Crossbreds (*n* = 27)		*P*
0.6%	0.5%	0.5%		0.96
**Average lambing rate at flock level**				
≤1.5 (*n* = 45)	>1.5 (n = 15)			*p*
0.5%	0.8%			0.034
**Control of reproduction**				
Intravaginal use of progestogen sponges (*n* = 13)	Administration of melatonin implants (*n* = 9)	No (*n* =38)		*p*
0.4%	0.5%	0.6%		0.26
**Feed origin**				
Homemade (*n* = 26)	Commercial (*n* = 34)			*p*
0.6%	0.5%			0.52
**Inclusion of roughage into the diet**				
Yes (*n* = 58)	No (*n* = 2)			*p*
0.6%	0.4%			0.77
**Intramammary administration of antibiotics at the end of the previous lactation period**				
Yes (*n* = 21)	No (*n* = 39)			*p*
0.6%	0.5%			0.96
**Nutritional modifications performed during the last stage of pregnancy**				
Yes (*n* = 28)	No (*n* = 32)			*p*
0.5%	0.6%			0.39
**Administration of anthelmintics at the end of pregnancy**				
Yes (*n* = 60)	No (*n* = 0)			*p*
0.5%	n/a			n/a
**Administration of anti-clostridial vaccination at the end of pregnancy**				
Yes (*n* = 60)	No (*n* = 0)			*p*
0.5%	n/a			n/a
**Administration of anti-mastitis vaccination at the end of pregnancy**				
Yes (*n* = 25)	No (*n* = 35)			*p*
0.5%	0.6%			0.25
**Experience of the farmer**				
0–10 years (*n* = 13)	11–20 years (*n* = 30)	>20 years (*n* = 17)		*P*
0.7%	0.3%	1.0%		<0.0001
**Presence of working staff in the farm**				
Yes (*n* = 39)	No (*n* = 21)			*p*
0.6%	0.5%			0.52

Table S6. Incidence risk of deaths of ewes during the 'at lambing' component of the peri-parturient period during a two-year study in 60 sheep farms in Greece, in accord with factors (*n* = 15) evaluated for potential association with these deaths.

**Management system applied in the flock**				
Intensive (*n* = 13)	Semi-intensive (*n* = 12)	Semi-extensive or extensive (*n* = 35)		*p*
0.1%	0.0%	0.1%		0.93
**Season of the year during which the lambing period took place**				
Winter (*n* = 5)	Spring (*n* = 0)	Summer (*n* = 19)	Autumn (*n* = 36)	*p*
0.1%	n/a	1.0%	0.0%	0.075
**Number of animals in the flock**				
≤200 ewes (*n* = 38)	200–500 ewes (*n* = 17)	>500 ewes (*n* = 5)		*p*
0.1%	0.1%	0.0%		0.29
**Breed of ewes**				
Greek pure-breed animals (*n* = 15)	Imported pure-breed animals (*n* = 18)	Crossbreds (*n* = 27)		*P*
0.1%	0.1%	0.0%		0.095
**Average lambing rate at flock level**				
≤1.5 (*n* = 45)	>1.5 (n = 15)			*p*
0.1%	0.0%			0.49
**Control of reproduction**				
Intravaginal use of progestogen sponges (*n* = 13)	Administration of melatonin implants (*n* = 9)	No (*n* =38)		*p*
0.0%	0.1%	0.0%		0.17
**Feed origin**				
Homemade (*n* = 26)	Commercial (*n* = 34)			*p*
0.0%	0.1%			0.40
**Inclusion of roughage into the diet**				
Yes (*n* = 58)	No (*n* = 2)			*p*
0.1%	0.0%			0.62
**Intramammary administration of antibiotics at the end of the previous lactation period**				
Yes (*n* = 21)	No (*n* = 39)			*p*
0.1%	0.0%			0.38
**Nutritional modifications performed during the last stage of pregnancy**				
Yes (*n* = 28)	No (*n* = 32)			*p*
0.1%	0.0%			0.69
**Administration of anthelmintics at the end of pregnancy**				
Yes (*n* = 60)	No (*n* = 0)			*p*
0.5%	n/a			n/a
**Administration of anti-clostridial vaccination at the end of pregnancy**				
Yes (*n* = 60)	No (*n* = 0)			*p*
0.5%	n/a			n/a
**Administration of anti-mastitis vaccination at the end of pregnancy**				
Yes (*n* = 25)	No (*n* = 35)			*p*
0.1%	0.1%			0.95
**Experience of the farmer**				
0–10 years (*n* = 13)	11–20 years (*n* = 30)	>20 years (*n* = 17)		*P*
0.0%	0.1%	0.0%		0.58
**Presence of working staff in the farm**				
Yes (*n* = 39)	No (*n* = 21)			*p*
0.0%	0.1%			0.035

Table S7. Incidence risk of deaths of ewes during the 'after lambing' component of the peri-parturient period during a two-year study in 60 sheep farms in Greece, in accord with factors (*n* = 15) evaluated for potential association with these deaths.

Management system applied in the flock

Intensive (*n* = 13)	Semi-intensive (*n* = 12)	Semi-extensive or extensive (*n* = 35)	*p*
0.3%	0.1%	0.2%	0.30

Season of the year during which the lambing period took place

Winter (*n* = 5)	Spring (*n* = 0)	Summer (*n* = 19)	Autumn (*n* = 36)	*p*
0.3%	n/a	0.3%	0.2%	0.48

Number of animals in the flock

≤200 ewes (*n* = 38)	200–500 ewes (*n* = 17)	>500 ewes (*n* = 5)	*p*
0.4%	0.1%	0.1%	0.022

Breed of ewes

Greek pure-breed animals (*n* = 15)	Imported pure-breed animals (*n* = 18)	Crossbreds (*n* = 27)	*P*
0.3%	0.1%	0.2%	0.19

Average lambing rate at flock level

≤1.5 (*n* = 45)	>1.5 (n = 15)	*p*
0.2%	0.4%	0.018

Control of reproduction

Intravaginal use of progestogen sponges (*n* = 13)	Administration of melatonin implants (*n* = 9)	No (*n* =38)	*p*
0.2%	0.4%	0.2%	0.079

Feed origin

Homemade (*n* = 26)	Commercial (*n* = 34)	*p*
0.2%	0.2%	0.70

Inclusion of roughage into the diet

Yes (*n* = 58)	No (*n* = 2)	*p*
0.2%	0.5%	0.28

Intramammary administration of antibiotics at the end of the previous lactation period

Yes (*n* = 21)	No (*n* = 39)	*p*
0.2%	0.2%	0.95

Nutritional modifications performed during the last stage of pregnancy

Yes (*n* = 28)	No (*n* = 32)	*p*
0.3%	0.1%	0.005

Administration of anthelmintics at the end of pregnancy

Yes (*n* = 60)	No (*n* = 0)	*p*
0.5%	n/a	n/a

Administration of anti-clostridial vaccination at the end of pregnancy

Yes (*n* = 60)	No (*n* = 0)	*p*
0.5%	n/a	n/a

Administration of anti-mastitis vaccination at the end of pregnancy

Yes (*n* = 25)	No (*n* = 35)	*p*
0.2%	0.2%	0.43

Experience of the farmer

0–10 years (*n* = 13)	11–20 years (*n* = 30)	>20 years (*n* = 17)	*P*
0.3%	0.1%	0.3%	0.074

Presence of working staff in the farm

Yes (*n* = 39)	No (*n* = 21)	*p*
0.2%	0.2%	0.72

Table S8. Summary of results of analysis for associations with peri-parturient deaths during a two-year study in 60 sheep farms in Greece, with the results of each year of the study considered separately.

**Occurrence of deaths: before lambing**	
**1st year of the study**	
Significant variables in the univariable analysis	*p*
Management system applied in the flock	<0.0001
Season of the year during which the lambing period took place	<0.0001
Experience of the farmer	<0.0001
Significant variables in the multivariable analysis	*p*
Management system applied in the flock	0.018
Season of the year during which the lambing period took place	0.046
Experience of the farmer	0.025
**2nd year of the study**	
Significant variables in the univariable analysis	*p*
Season of the year during which the lambing period took place	<0.0001
Experience of the farmer	0.022
Most significant variable in the multivariable analysis	*p*
Season of the year during which the lambing period took place	0.095
**Occurrence of deaths: at lambing**	
**1st year of the study**	
Significant variables in the univariable analysis	*p*
Most significant variable in the multivariable analysis	*p*
Presence of working staff in the farm	0.28
**2nd year of the study**	
Significant variables in the univariable analysis	*p*
Season of the year during which the lambing period took place	0.001
Breed of ewes	0.022
Control of reproduction	0.0005
Presence of working staff in the farm	0.002
Significant variable in the multivariable analysis	*p*
Presence of working staff in the farm	0.030
**Occurrence of deaths: after lambing**	
**1st year of the study**	
Significant variables in the univariable analysis	*p*
Number of animals in the flock	0.004
Breed of ewes	<0.0001
Control of reproduction	0.002
Nutritional modifications performed during the last stage of pregnancy	0.002
Experience of the farmer	0.022
Most significant variable in the multivariable analysis	*p*
Control of reproduction	0.063
**2nd year of the study**	
Significant variables in the univariable analysis	*p*
Number of animals in the flock	0.046
Nutritional modifications performed during the last stage of pregnancy	0.031
Significant variable in the multivariable analysis	*p*
Nutritional modifications performed during the last stage of pregnancy	0.046
